# A group-theoretical notation for disease states: an example using the psychiatric rating scale

**DOI:** 10.1186/1742-4682-9-28

**Published:** 2012-07-09

**Authors:** Jitsuki Sawamura, Shigeru Morishita, Jun Ishigooka

**Affiliations:** 1 Department of Psychiatry, Tokyo Women’s Medical University, Tokyo, Japan; 2 Depression Prevention Medical Center, Kyoto Jujo Rehabilitation Hospital, Kyoto, Japan

**Keywords:** Group theory, Modulo operation, Severity assessment, BPRS, Notation

## Abstract

**Background:**

While many branches of natural science have embraced group theory reaping enormous advantages for their respective fields, clinical medicine lacks to date such applications. Here we intend to explain a prototypal model based on the postulates of groups that could have potential in categorizing clinical states.

**Method:**

As an example, we begin by modifying the original ‘Brief Psychiatric Rating Scale’ (BPRS), the most frequently used standards for evaluating the psychopathology of patients with schizophrenia. We consider a presumptively idealized (virtually standardized) BPRS (denoted BPRS-I) with assessments ranging from ‘0’ to ‘6’ to simplify our discussion. Next, we introduce the modulo group Z_7_ containing elements {0,1,2,…,6} defined by composition rule, ‘modulo 7 addition’, denoted by *. Each element corresponds to a score resulting from grading a symptom under the BPRS-I assessment. By grading all symptoms associated with the illness, a Cartesian product, denoted A_j,_ constitutes a summary of a patient assessment. By considering operations denoted A_(j→k)_ that change state A_j_ into state A_k_, a group M (that itself contains A_j_ and A_k_ as elements) is also considered. Furthermore, composition of these operations obey modulo 7 arithmetic (i.e., addition, multiplication, and division). We demonstrate the application with a simple example in the form of a series of states (A_4_ = A_1_*A_(1→2)_*A_(2→3)_*A_(3→4)_) to illustrate this result.

**Results:**

The psychiatric disease states are defined as 18-fold Cartesian products of Z_7_, i.e., Z_7_^×18^ = Z_7_×…×Z_7_ (18 times). We can construct set G ≡ {a_(m)i_| m = 1,2,3,…(the patient’s history of the i-th symptom)} and M ≡ {A_m_ | A_m_ ∈ Z_7_^×18^ (the set of all possible assessments of a patient)} simplistically, at least, in terms of modulo 7 addition that satisfies the group postulates.

**Conclusions:**

Despite the large limitations of our methodology, there are grounds not only within psychiatry but also within other medical fields to consider more generalized notions based on groups (if not rings and fields). These might enable through some graduated expression a systematization of medical states and of medical procedures in a manner more aligned with other branches of natural science.

## Background

Group theory is a branch of abstract algebra developed to classify and study abstract concepts involving symmetry [1-3]. In particular, in the 20^th^ century, it formed one of the cornerstones of mathematical methods in physics where group representation theory provided the setting to quantum fields (i.e., Poincare group [2,4]) and special relativity (i.e., Lorentz group [5-7]). In general, when group theory is used in physics, it is usually in the role of describing geometrical or dynamical symmetries of phenomena under consideration. For example, all the equations of classical Newtonian mechanics are invariant under Galilean transformations. In solid-state physics, the symmetry groups of crystals play a central role, and parts of chemistry concerning molecular systems can be cast in terms of group-theoretic expressions [3]. Cosmological, stellar, and atomic particle abstractions can also be expressed quite powerfully in terms of group theory [6,8]. From social anthropology, ‘*Les structures élémentaires de la parenté*’ was written by C. Lévi-Strauss in 1949 based on André Weil’s study, ‘Algebraic Study of Certain Types of Marriage (Murngin System)’, that initiated a series of articles on the mathematical treatment of marriage rules [9]. We emphasize though that we are not bringing structuralism ideas into clinical psychiatry in applying our group-theoretic approach. Researchers in the field of molecular biology understand the cell cycle and genetic activities from the standpoint of group operations [10]. Underlying group theory’s usefulness in the various branches of natural science is the notion that the group itself serves as the configuration space for the system under consideration.

The medical field embraces many branches of natural science (physics and chemistry) as well as others such as evolutionism, public health, (molecular) biology, genetics, biological engineering, and some parts of mathematics. However, medicine is not the mere combination of these branches. Also, over time, delineating medicine from these is becoming harder. To our knowledge, medicine unfortunately has not been systematized enough in general, and has not attained a level of sophistication that links it directly with other fields of natural science. As is well known, the present structure and/or schematization of medicine is too complex to treat effectually in a rational manner. The descriptive nature of medicine depends on classifying medical states of patients, and the results of treatments might be sufficiently non-optimal to be unable to work out a reasonably scientific system. That might yield more than a little degree of futility. In our view, one reason for that is the lack in medical science of a group-theory systematics, in particular an effective notational style. Group theory provides a framework to abstract simple models from real-world systems, and to analyze those models in designing new systems and in making new predictions. For that reason, we believe that group theory might be crucial, and upgrading medical procedures and refine its procedural style, particularly in terms of description and manipulation, even partially, is necessary to refine the medical field for the future. We envisage that it would reduce the magnitude of data and/or handling requirements of patient medical records.

At any rate, by some means or other, we need to formulate a model that would be based on some group-theorized rule, even if it is incomplete and insufficient to meet our desires at this early stage.

Using as an example from clinical medicine a psychiatric evaluation scale that scores disease severity, we describe below a prototypal model based on group theory ideas that could provide an operational structure and play an important role in treatment programs. With such a model, we hope to infer its potential to categorize clinical states. In a similar manner, we believe that this technique could also apply to cancer stages, describing the severity based on the extent of the tumor and spread within the body. In addition, we demonstrate the application with a simple example that might be useful in imagining a potentially descriptive style in medicine for the future. Combined with the use of personal computers and other electronic tools, we see a move towards a future of optimally systemized medicine.

The main idea behind our method has been considerably influenced by Naganuma’s “Theory of operation matrix” proposal [11], although the core concept has been published only in Japanese at present. There, wide-ranging types of physical quantities are combined to form an approximately tautologized vector ‘x’ along with various information. These include limitations associated with a certain model that are able to be displayed by a square matrix (Operation Matrix) [A] that acts at ‘x’ within a specified time interval either as ‘x(t + Δt) = [A]x(t)’ or ‘x(t + n·Δt) = [A^n^x(t)’ where t is the time and Δt a minimal time interval. It seems to us that our model is a specific example of Naganuma’s model.

## Model assumptions

To illustrate our concept in a more simplified style, we focus on schizophrenia and its psychiatric evaluation scale as an example from clinical medicine. By making use of these materials, we intend to develop our discussion in group-theorizing disease states within clinical medicine generally.

### §1. Group-theory model to classify psychiatric disease states via a presumptively idealized ‘Brief Psychiatric Rating Scale’

Conventionally, the ‘Brief Psychiatric Rating Scale’ (BPRS) [12] is the more frequently used standard for evaluating the psychopathology of patients with schizophrenia, not only in clinical practice but also in clinical research. However, although it applies to every psychiatric state, the scale also includes non-disease states and hence applies to all patients whether suffering from schizophrenia or not. The BPRS provides an assessment of 18 symptoms (somatic concern, anxiety, emotional withdrawal, conceptual disorganization, guilt, tension, bizarre behavior, grandiosity, depressed mood, hostility, suspiciousness, hallucinations, motor retardation, uncooperativeness, unusual thought content, blunted affect, excitement and disorientation). An assessment is clearly defined as scores for all symptoms have the same classification: (1 (not present), 2 (very mild), 3 (mild), 4 (moderate), 5 (moderately severe), 6 (sever) and 7 (extremely severe), with ‘0’ meaning “not assessed”). Psychiatrists assess their patients for symptoms associated with schizophrenia, rate the severities of each symptom, ordinarily from 1 to 7, and sum to obtain a total score from 18 to 126. Ideally, the BPRS score indicates the severity of the psychiatric disease state, although this assumption has weak theoretical basis. Scoring across symptoms might not be equally standardized; the BPRS is to date not determined in a sufficiently reasonable and rigorous way.

To simplify discussions, we next consider a presumptively idealized (virtually standardized) BPRS, called “BPRS-I”, that still includes all 18 symptoms. Scoring is the same for all symptoms but with modified range (0 for not present to 6 for extremely severe; an average score would be 3). Of course, assessment assumes ideal objectivity. Each symptom is assessed against a golden standard with perfect linearity of scale, and having the necessary and sufficient grades; although this assumption also seems to have only a weak theoretical premise. That aside, we believe that this kind of simplification is unavoidable. The necessary trade-off sacrifices rigor to some extent in exchange for constructing our theoretical model. Moreover, all symptoms are assumed to be graded over a 7-point scale to further simply discussions.

We now turn attention to describing the mathematical aspects that will help to understand content discussed later. In general, we can divide any number ‘a’ by ‘7’ and find its remainder, i.e., one of the integers in set Z_7_≡{0, 1, 2, 3, 4, 5, 6}. This is called ‘modulo 7 reduction’. Now, if we add two numbers and perform the same operation, we get also a number with remainder in Z_7_. This rule for adding among integers is called ‘modulo 7 addition’. We can also replace ‘7’ by any natural number ‘m’ and do exactly the same thing; Z_m_ now denotes the set {0, 1, 2, …, m − 1}. Any integer N can then be mapped to Z_m_ by dividing by ‘m’ and taking the remainder, this is called ‘modulo m reduction’, and the resulting remainder is written N mod m.

In addition, the Cartesian product of two sets X and Y, denoted ‘X × Y’, can be defined by

(1) $$\text{X}\times \text{Y}=\{\left(\text{x},\text{y}\right)|\text{x}\in \text{X and y}\in \text{Y}\}\text{.}$$

For example, let $\text{X}=\left\{\text{a},\text{b},\text{c},\text{d}\right\}$ and let $\text{Y}=\left\{1,2\right\}\text{,}$

(2) $$\text{X}\times \text{Y}=\left\{\left(\text{a},1\right),\left(\text{b},1\right),\left(\text{c},1\right),\left(\text{d},1\right),\left(\text{a},2\right),\left(\text{b},2\right),\left(\text{c},2\right),\left(\text{d},2\right)\right\}\text{.}$$

Hence, by using Z_m_ as X and Y, we can define the set ‘Z_m_ × Z_m_’, and a large number of combinations of ‘Z_m_’s are also composable as ‘Z_m_ × Z_m_ × … × Z_m_’ [2].

In this article, we consider the case m = 7; that is ‘Z_7_’ and ‘Z_7_ × Z_7_ × … × Z_7_’. There are two reasons we select the natural number ‘7’; one is that ‘7’ is the grade associated with each individual symptom in the BPRS (or BPRS-I) considered above; the other is that ‘7’ is a prime number that is convenient in modeling our concept. A prime number is often used to ease the computational burden of computing arithmetic operations modulo prime numbers, as will become apparent below [13,14].

We now define modulo addition to complete the description of Z_m_. From numbers ‘a’ and ‘b’ that belong to Z_m_, we can compute a new number called a + b (mod m) that is also in Z_m_, as follows: compute a + b, divide by m and let a + b mod m be the remainder. Since we divide by m, the remainder will always be in Z_m_. For instance, with m = 7, 2 + 3 (mod 7) = 5 (mod 7), 5 + 2 (mod 7) = 0 (mod 7), 4 + 5 (mod 7) = 2 (mod 7).

Modulo subtraction follows similar results as for addition because the result that ‘(a-b) mod n = a mod n - b mod n’ is modulo addition by using the result ‘(−b) mod n = −b mod n’.

Modulo multiplication is also a possible operation that applies in our context. First, modulo multiplication a × b (mod m) is by definition the remainder when dividing a × b by m. For example, 4 × 5 (mod 7) = 6 (mod 7). With element ‘a’, ‘a × Z_7_ (mod 7) = Z_7_ (mod 7)’ is true.

Modulo division by a (mod 7) is also definable (See Appendix A for details).

As a further step, we assume that the state of a certain patient evaluated by using the BPRS-I can be described as a vector composed of scores from all 18 symptoms of the BPRS (BPRS-I). Strictly, our subsequent discussions hold only under the tentative assumption that increments associated with the assessment scale (here, the BPRS-I) are equal, and arbitrary scores can be summed as if these were comparable, reflecting exactly true objective values corresponding to the disease states. Again, there is no evidence to support this assumption, but we believe the simplification is necessary for similar reasons given earlier.

Next, let ‘a_(1)i_∈Z_7_’_,_ i.e., 0 ≤ a_(1)i_ ≤ 6, be a patient’s initial assessment of the i-th symptom of schizophrenia; here i = 1,.,n, with ‘n’ a natural number taking value n = 18 corresponding to the number of symptoms assessed. Using the set of ‘a_(1)i_’s, the complete initial assessment ‘A_1_’ is written in a vector-like form:

(3) $${\text{A}}_{1}=[{\text{a}}_{\left(1\right)1}|{\text{a}}_{\left(1\right)2}|{\text{a}}_{\left(1\right)3}|\ldots |{\text{a}}_{\left(1\right)\text{i}}|\ldots |{\text{a}}_{\left(1\right)\text{n}-1}|{\text{a}}_{\left(1\right)\text{n}}]\text{.}$$

A_1_ is the n-tuple product of components with the additional meaning that these obey modulo 7 arithmetic; to denote this, we add ‘mod 7’ at the end of the vector:

(4) $${\text{A}}_{1}=[{\text{a}}_{\left(1\right)1}|{\text{a}}_{\left(1\right)2}|{\text{a}}_{\left(1\right)3}|\ldots |{\text{a}}_{\left(1\right)\text{i}}|\ldots |{\text{a}}_{\left(1\right)\text{n}-1}|{\text{a}}_{\left(1\right)\text{n}}]\left(\text{mod 7}\right)\text{.}$$

(See Appendix B for an example.)

The underlying structure of each A_m_ is the 18-fold Cartesian product of Z_7_, i.e., Z_7_^×18^ = Z_7_×…×Z_7_ (18 times). The model we intend to propose is based on the modulo groups Z_p_ (p integer) with p = 7. Note that the state ‘E’, composed of all components with values ‘0’ (not present)’, represents a completely healthy state within the BPRS-I:

(5) $$\text{E}\equiv [0\left|0\right|0|\ldots \left|0\right|\ldots |0|0]\left(\text{mod 7}\right)\text{.}$$

We need to give the rules to interpret these. While also being states associated with a patient’s assessments, the states ‘A_m_’ can be interpreted in two ways. One is as absolute states that express the severity of the symptoms for a specified patient. The second is as an operator that acts on healthy state E and produces disease state ‘A_m_’ (see Appendix C for details). In this regard, we shall assume that ‘A_m_’ is interpreted by the former meaning when we consider disease states of a specified patient without provisory context.

Likewise, by considering the set of all possible assessment states A_m_, the operation linking the pair A_j_ and A_k_ can be expressed as a unique A_m_ obtained under modulo 7 addition. This operation must be performed independently for each and every individual component as below:

(6) $$\begin{array}{l} {\text{A}}_{\text{j}}\left(\text{mod 7}\right)+{\text{A}}_{\text{k}}\left(\text{mod 7}\right)=\left\{{\text{A}}_{\text{j}}+{\text{A}}_{\text{k}}\right\}\;\left(\text{mod 7}\right)\\ =[{\text{a}}_{\left(\text{j}\right)1}|{\text{a}}_{\left(\text{j}\right)2}|{\text{a}}_{\left(\text{j}\right)3}|\ldots |{\text{a}}_{\left(\text{j}\right)\text{i}}|\ldots |{\text{a}}_{\left(\text{j}\right)\text{n}-1}|{\text{a}}_{\left(\text{j}\right)\text{n}}]\left(\text{mod 7}\right)\\ +[{\text{a}}_{\left(\text{k}\right)1}|{\text{a}}_{\left(\text{k}\right)2}|{\text{a}}_{\left(\text{k}\right)3}|\ldots |{\text{a}}_{\left(\text{k}\right)\text{i}}|\ldots |{\text{a}}_{\left(\text{k}\right)\text{n}-1}|{\text{a}}_{\left(\text{k}\right)\text{n}}]\left(\text{mod 7}\right)\\ =[{\text{a}}_{\left(\text{j}\right)1}+{\text{a}}_{\left(\text{k}\right)1}|{\text{a}}_{\left(\text{j}\right)2}+{\text{a}}_{\left(\text{k}\right)2}|\ldots |{\text{a}}_{\left(\text{j}\right)\text{i}}+{\text{a}}_{\left(\text{k}\right)\text{i}}|\ldots |{\text{a}}_{\left(\text{j}\right)\text{n}-1}+{\text{a}}_{\left(\text{k}\right)\text{n}-1}|{\text{a}}_{\left(\text{j}\right)\text{n}}+{\text{a}}_{\left(\text{k}\right)\text{n}}]\left(\text{mod 7}\right)\\ \;(\text{j,k = 1,2,3,…; positive integers}) \end{array}$$

Based on these notions, we are able to define set G consisting of a_(m)i_ (i-th component of the state A_m_), with composition mod 7 addition:

(7) $$\begin{array}{l} \text{G}\equiv \{{\text{a}}_{\left(\text{m}\right)\text{i}}|\text{ m}=1,2,3,\ldots \}\left(\text{the patient}’\text{s history of the i}-\text{th symptom}\right)\\ \left(\text{i}=1,\ldots ,\text{n},\text{ n}=18,\text{ m a natural number}\right) \end{array}$$

Therefore, we can consider a set ‘M = Z_7_^×18^ = Z_7_×…×Z_7_ (18 times)’ as the set of all possible assessments with the following extended rule of composition based on modulo 7 addition:

(8) $$\text{M}\equiv \{{\text{A}}_{\text{m}}|{\text{A}}_{\text{m}}є{\text{Z}}_{7}{{}^{x}}^{1}{}^{8}\}$$

(m; a natural number, the number of distinct M is 7^18^ (≡W)).

Under these conditions, we confirm that, as both ‘G’ and ‘M’ are additive groups with composition ‘modulo7 addition’ (both denoted by *), these sets satisfy the following three conditions:

For set G:

Іa) Associativity: for any i-th component a_(j)i_ (∈ G), the relation

(9) $$\left({\text{a}}_{\left(\text{j}\right)\text{i}}*{\text{a}}_{\left(\text{k}\right)\text{i}})*{\text{ a}}_{\left(\text{l}\right)\text{i}}={\text{a}}_{\left(\text{j}\right)\text{i}}*({\text{a}}_{\left(\text{k}\right)\text{i}}*{\text{ a}}_{\left(\text{l}\right)\text{i}}\right)$$

that is, {(a_(j)i_ + a_(k)i_) + a_(l)i_} (mod 7) = {a_(j)i_ + (a_(k)i_ + a_(l)i_)} (mod 7)

(j, k, l = 1,2,3,…; positive integers), holds.

IIa) Identity: for any i-th component a_(j)i_ (∈ G), there exists an identity element e (= 0) ∈ G, such that a_(j)i_*e = e*a_(j)i_ = a_(j)i_

that is {a_(j)i_ + e} (mod 7) = {e + a_(j)i_} (mod 7) = a_(j)i_ (mod 7), holds.

IIIa) Inverse: for any i-th component a_(j)i_ (∈ G), there exists a unique element (a_(j)i_) ∈ G, such that a_(j)i_*(a_(j)i_) = (a_(j)i_)* a_(j)i_ = e (= 0)

that is, {a_(j)i_ + (a_(j)i_)} (mod 7) = {(a_(j)i_) + a_(j)i_} (mod 7) = e (mod 7)

for each individual component. By choosing “(a_(j)i_) = 7- a_(j)i_ (mod 7)”

(in this regard 7 (mod 7) = 0 (mod 7)), the above relation would be satisfied.

Therefore, set G =( Z_7_,*) satisfies the group postulates and has order 7 [1,2]. Likewise for set M; because set M is a Cartesian product of set G, M also satisfies the group postulates (Details given in Appendix D). Therefore, as M = (Z_7_^×18^,*), the order of M is 7^18^ (=W).

However, because Z_p_ (p integer) satisfies the group postulates and, consequently, G and M are N- and n-tuple Cartesian product groups, respectively, these must also satisfy the group postulates. Indeed, all Z_p_ groups are Abelian, meaning ‘a*b = b*a’ [1,2]. The above results depend upon modulo addition being performed independently for each component.

We can now describe how we can interpret the change in state between two states, ‘A_j_ → A_k_’ (mod 7). By defining the difference ‘A_(j→k)_ (mod 7) = A_k_ (mod 7)- A_j_ (mod 7) = {A_k_ - A_j_} (mod 7)’, which expresses the direct change between the two states of illness, A_k_ can then be expressed using ‘*’ as follows:

(10) $${\text{A}}_{\text{j}}*{\text{ A}}_{\left(\text{j}\to \text{k}\right)}={\text{ A}}_{\text{j}}\left(\text{mod }7\right)+{\text{ A}}_{\left(\text{j}\to \text{k}\right)}\left(\text{mod }7\right)=\left\{{\text{A}}_{\text{j}}+{\text{ A}}_{\left(\text{j}\to \text{k}\right)}\right\}\;\left(\text{mod }7\right)={\text{ A}}_{\text{k}}\left(\text{mod 7}\right)\text{.}$$

In other words, the operation A_(j→k)_ (mod 7) acting on the state A_j_ (mod 7) produces state A_k_ (mod 7) (see Appendix E for further description; a concrete example is also given in Appendix F).

For these reasons, we are able in the above to perform additive operations as modulo 7 addition and we can treat the states described via the vector-like expressions (Cartesian products) using the presumptively idealized BPRS (BPRS-I) within a group theory context. Although the practical implications of this model have yet to be explored, we believe we are able to extend and make efficient use of the idea in more generalized medical settings.

According to these discussions, the states A_j_ classify the psychiatric states of patients within the BPRS-I. We interpret A_j_^-1^ (the inverse element of A_j_) as meaning the ideally desirable patient recovery that includes natural changes over the course of time as well as treatments themselves that the schizophrenic patients (or others) need to undergo. Operationally, ‘A_j_*(A_j_)^-1^ = E (= A_0_)’ returning the patient to a completely healthy state accomplished by operator (A_j_)^-1^ = A_(j→0)_ when acting on A_j_.

### §2. Notational development of disease states from ‘group’ to ‘ring’ or ‘field’ via modulo multiplication and division

As illustrated above, multiplication and division with respect to ‘A_j_’ is definable (A demonstration is presented in Appendix G). Then, modulo 7 multiplication can be defined as an operation on G and M.

In addition to the group postulates determined for G and M, we verify that the subsequent three conditions, ‘commutativity’, ‘associativity’, and ‘distributivity’, hold for all ‘a_(j)i_’s (∈ G) and ‘A_j_’s (∈ M) (See Appendix H for proof).

Therefore, G and M satisfy the composition rules for ‘rings’ [1,2].

Moreover, with the existence of additive inverses and multiplicative inverses, G and M satisfy separately the conditions for ‘fields’ [1,2].

Again, the reasons why multiplication and division as modulo operations on M can be defined is that the number ‘7’ is prime; the use of a prime modulus ensures that the multiplicative inverse A_j_^-1^ exists for any A_j_[13,14]. If, for example, the composition law of M were to be defined modulo ‘10’, it would be convenient to understand such models within the base-10 system where analysis would be more effective. However, given this decimal system (modulo 10), only addition (or subtraction) is possible as multiplication and division cannot be uniquely defined because ‘10’ is not prime; the uniqueness of ‘A_j_^-1^’ is lost.

On the bases of these results, if the (j-1)-th state A_j-1_ changes into (j)-th state A_j_, the operation inducing this change would require ‘a_(j-1→j)i_’s, calculated by subtracting a_(j-1)i_ from a_(j)i_ retrospectively after the regular determinations of each a_(k)i_ (k = 1,…,j). We then write

(11) $${\text{A}}_{\left(\text{j}-1\to \text{j}\right)}=[{\text{a}}_{\left(\text{j}-1\to \text{j}\right)1}|{\text{a}}_{\left(\text{j}-1\to \text{j}\right)2}|{\text{a}}_{\left(\text{j}-1\to \text{j}\right)3}|\ldots |{\text{a}}_{\left(\text{j}-1\to \text{j}\right)\text{i}}|\ldots |{\text{a}}_{\left(\text{j}-1\to \text{j}\right)\text{n}-1}|{\text{a}}_{\left(\text{j}-1\to \text{j}\right)\text{n}}]\left(\text{mod }7\right)$$

where j = 2,…,m, with m a natural number (n is the number of components).

Thus, if a course of a treatment comprises m stages, ‘A_1_ → A_2_ → A_3_ → A_4_…A_m-2_ → A_m-1_ → A_m_’, the terminating state of this series A_m_ can be expressed recursively as the combination of the initial state A_1_ and operations A_(j-1→j)_ (these being also elements of M) with modulo 7 arithmetic (addition, subtraction, multiplication and division denoted collectively by ‘†’) in the following way:

(12) $${\text{A}}_{\text{m}}={\text{A}}_{1}†{\text{A}}_{\left(1\to 2\right)}†{\text{A}}_{\left(2\to 3\right)}†{\text{A}}_{\left(3\to 4\right)}†\ldots †{\text{A}}_{\left(\text{m}-2\to \text{m}-1\right)}†{\text{A}}_{(\text{m}-1}{}_{\to \text{m})}\text{,}$$

where all operations ‘A_(j-1→j)_’ (j = 2,…,m) would be determined retrospectively using the already confirmed states ‘A_1_, A_2_, …, A_m_’. Here ‘†’ could be any one of the arithmetic operations. In other words, all states A_i_ belong to field M, and implies that all such states are connected in a series of transitions defined by operations in M; all elements are then closed within M under modulo 7 arithmetic.

Now, as a first step, if we use solely modulo 7 addition ‘*’, the state A_m_ can be expressed recursively as follows:

(13) $${\text{A}}_{\text{m}}={\text{A}}_{1}*{\text{A}}_{\left(1\to 2\right)}*{\text{A}}_{\left(2\to 3\right)}*{\text{A}}_{\left(3\to 4\right)}*\ldots *{\text{A}}_{\left(\text{m}-2\to \text{m}-1\right)}*{\text{A}}_{(\text{m}-1}{}_{\to \text{m})}\text{.}$$

Naturally, this can be confirmed easily using simple examples. If we are given states

(14) $$\begin{array}{c} {\text{A}}_{1}=\left[1\left|4\right|0\text{|}\ldots \left|3\right|\ldots \left|6\right|1\right]\left(\text{mod 7}\right),\\ {\text{A}}_{2}=\left[2\left|6\right|3\text{|}\ldots \left|1\right|\ldots \left|0\right|5\right]\left(\text{mod 7}\right),\\ {\text{A}}_{3}=\left[4\left|0\right|5\text{|}\ldots \left|4\right|\ldots \left|3\right|6\right]\left(\text{mod 7}\right),\\ {\text{A}}_{4}=\left[0\left|2\right|3\text{|}\ldots \left|6\right|\ldots \left|2\right|4\right]\left(\text{mod 7}\right)\text{,} \end{array}$$

then the series of state-changes for ‘A_1_ → A_2_ → A_3_ → A_4_ (m = 4)’ can be deduced:

(15) $$\begin{array}{l} \begin{array}{l} {\text{A}}_{\left(1\to 2\right)}\left(\text{mod }7\right){\text{ = A}}_{2}\left(\text{mod }7\right){\text{ - A}}_{1}\left(\text{mod }7\right)\text{ = }\left\{{\text{A}}_{2}{-\text{ A}}_{1}\right\}\;\left(\text{mod }7\right)\\ \;\text{=}[2-1\left|6-4\right|3-0\text{|…}\left|1-3\right|\text{…}\left|0-6\right|5-1]\left(\text{mod }7\right)\\ \;\text{=}[1\left|2\right|3\text{|…}\left|5\right|\text{…}\left|1\right|4]\left(\text{mod }7\right), \end{array}\\ \begin{array}{l} \begin{array}{l} {\text{A}}_{\left(2\to 3\right)}\left(\text{mod }7\right)\text{ = }\left\{{\text{A}}_{3}{-\text{ A}}_{2}\right\}\;\left(\text{mod }7\right)\\ \;\text{=}[4-2\left|0-6\right|5-3\text{|…}\left|4-1\right|\text{…}\left|3-0\right|6-5]\left(\text{mod }7\right) \end{array}\\ \;\text{=}[2\left|1\right|2\text{|…}\left|3\right|\text{…}\left|3\right|1]\left(\text{mod }7\right),\\ {\text{A}}_{\left(3\to 4\right)}\left(\text{mod }7\right)\text{ = }\left\{{\text{A}}_{4}{-\text{ A}}_{3}\right\}\;\left(\text{mod }7\right)\\ \begin{array}{l} \;\text{=}[0-4\left|2-0\right|3-5\text{|…}\left|6-4\right|\text{…}\left|2-3\right|4-6]\left(\text{mod}\;7\right)\\ \;\text{=}[3\left|2\right|5\text{|…}\left|2\right|\text{…}\left|6\right|5]\left(\text{mod}\;7\right)\text{.} \end{array} \end{array} \end{array}$$

The terminating state of A_4_ can be recalculated recursively:

(16) $$\begin{array}{l} {\text{A}}_{4}{\text{ = A}}_{1}{\text{*A}}_{\left(1\to 2\right)}{\text{*A}}_{\left(2\to 3\right)}{\text{*A}}_{\left(3\to 4\right)}\left(\text{mod }7\right)\\ {\text{= A}}_{1}{\text{ + A}}_{\left(1\to 2\right)}{\text{ + A}}_{\left(2\to 3\right)}{\text{ + A}}_{\left(3\to 4\right)}\left(\text{mod }7\right)\\ \text{=}[1\left|4\right|0\text{|…}\left|3\right|\text{…}\left|6\right|1]\text{ + }[1\left|2\right|3\text{|…}\left|5\right|\text{…}\left|1\right|4]\\ \text{+}[2\left|1\right|2\text{|…}\left|3\right|\text{…}\left|3\right|1]+[3\left|2\right|5\text{|…}\left|2\right|\text{…}\left|6\right|5]\left(\text{mod }7\right)\\ \text{= [1 + 1 + 2 + }3\left|4\text{ + 2 + 1 + }2\right|0\text{ + 3 + 2 + 5|…}\left|3\text{ + 5 + 3 + }2\right|\text{…}\left|6\text{ + 1 + 3 + }6\right|1\text{ + 4 + 1 + }5]\left(\text{mod }7\right)\\ \text{= }[0\left|2\right|3\text{|…}\left|6\right|\text{…}\left|2\right|4]\left(\text{mod }7\right) \end{array}$$

## Results

As seen above, our example from within clinical medicine of psychiatric disease states are definable in the style of an 18-fold Cartesian product of Z_7_, i.e., Z_7_^×18^ = Z_7_×…×Z_7_ (18 times). We are able to construct in a simple manner set G ≡ {a_(m)i_| m = 1,2,3,…(the patient’s history of the i-th symptom)} and M ≡ {A_m_ | A_m_ ∈ Z_7_^×18^ (the set of all possible assessments of the patients)}, adding composition rule ‘modulo 7 addition’ so that G and M satisfy the group postulates.

## Discussion

In the “Model Assumptions” section, we gave a simple description of our ideas that might apply in classifying psychiatric states using group-theory notions. There, several samples were given describing changes in clinical diagnosis with/without interventional treatment. We took into account the severity of the 18 symptoms associated with schizophrenia by devising a presumptively idealized evaluation scale based on the BPRS-I to help simplify our discussions. By setting up seven grades of severity, indexed from 0 to 6, we regarded the assessed patient scores ‘A_j_’ = [a_(j)1_|…|a_(j)18_] of schizophrenia not only as patient states but as operations that act on patient states under modulo 7 addition. The associated group structure Z_7_ is finite, being composed of elements {0,1,2,3,4,5,6}, the group G is {a_(m)i_| m = 1,2,3,…}, and the group M is {A_m_ | A_m_ ∈ Z_7_^×18^}, the focal structure of our method, being composed as an 18-tuple combination of elements of G. To broaden the applicability of group M, we added modulo 7 multiplication and division to extend G as a ring or field. The conjecture is that, with modulo 7 arithmetic, M could potentially be the construct applicable in clinical psychiatry in particular and medicine in general.

We envisage something along the following lines. If algorithms are established adequately, we see advantages in monitoring patients’ progress throughout the course of the psychiatric condition, particularly when specific medication is prescribed. Ordinal ratings or scoring from diagnostic evaluations are merely a series of combinations of scores ‘A_1_,A_2_, …,A_m_’. However, if these are expressed as in (6), we are then able to trace a clinical course easily and recognize the changes that occur at successive evaluations. This change in perspective is, we infer, one of the novelties of our model. For this reason, transitional expressions, as in (6), clearly emphasize a patient’s clinical progress from an initial state A_1_ to a current state A_m_. We believe that this viewpoint might enable us to understand the progressive states of each patient over stepwise intervals. Moreover, we expect unknown advantages might exist in this kind of sophisticated handling or monitoring of disease states that algebra, and particularly groups, rings, and fields, affords.

In terms of standardizing the application of this model, the rules for determining the scores might be various. For example, comparison between particular components at different moments could be possible, thereby two different states of a patient with specified symptoms could be assessed for effectiveness of a specified medication administered over some time period; e.g., we can compare pre- and post-administering states of a patient given sertraline 25 mg/d over a 14 day period. Alternatively, a plurality of components could be assessed during changes over the same period to overview the evolution of various symptoms following a certain treatment given to a patient. In this regard, we note that changes in the components also include changes in the natural course of the illness independent of whether treatment is given or not. For these reasons, we presume that, given a rigorous methodology concerning operations to be conducted within this model, the algebra perspective as captured by modulo arithmetic might be of advantage in recording and handling large amounts of data, i.e., the many patient histories and treatments. Although this description might not be expected for the present to produce clinical prognoses, we believe that this can be a goal for the future if foundations are prepared rigorously.

We conjecture that there might be grounds to expect that our model might be useful in compressing the vast amounts of clinical data, especially as personal computers are in daily use. Interestingly, symptom gradings other than the BPRS (BPRS-I) are also definable. For instance, the severity of ‘headache’, ‘nausea’, ‘hypernatremia’, ‘hypokalemia’, ‘hypercreatinemia’ and ‘hyperthyroidism’ might be Z_7_-gradable in future medical practice (namely, j = 1,.,m with m the number of assessment sessions, i = 1,.,n with n the number of symptoms related to a given patient’s illness). Moreover, TMN classification of malignant tumors could be also described in the same way (e.g., Stage: T0 through T6 to represent the development from absence of tumors to small tumors, to spreading and/or invasive primary tumors; Stage: M0 through M6 to represent distant metastasis). The model we proposed might be applicable to any set of symptoms and clinical findings that is able to be quantified in a similar way. Additionally, if symptoms are complementary (e.g., ‘hypernatremia’ and ‘hyponatremia’), then one or other should be ‘0’. If severity can be linearly graded and ideally standardized through rigorous optimized methods (e.g., hypernatremia scoring ranges of 0: 135−145, 1: 146−149, 2: 150−153, 3:154−157, 4:158−164, 5:165−170, 6: ≥ 170 mEq/l), a generalized application like the International Classification of Diseases 10-th Revision (ICD-10) might be possible for any disease once symptoms (clinical findings) have been correlated with an indexing number ‘i’. To broaden the ICD-10-like utilities, future studies in line with our methods would be desirable. Naturally, determining an optimal grading of severity of symptoms at the assessment stage is a crucial issue if this model is to be useful in any way.

Most of all, one of our particular concerns about the model is its applicability. The treatment of a group by its matrix representation has been extensively studied [15]. Thereby, idealistically, using suitable methods, the prescribed state of illness is presumed to display appropriate group theoretic properties investigable by theorems of group theory. If, in particular, the severities of many symptoms of patients can be expressed in a square-matrix form, matrix functions might be of use in calculating specific universal values (e.g., the quadratic form of matrix) that quantify illness characteristics. Also, eigenvalues of the matrix might be of advantage in understanding illness pathology or in planning treatments. In addition, notions from other fields might be of possible applicability to the present model; for instance, the Chinese remainder theorem, geometric algebra, algebraic coding theory, and matrix group, where there might be potential affinities between these and our present model [1,2].

Intriguingly, partial combinations of ‘a_(j)i_’s such as Å_j_ = [a_(m)1_|a_(m)4_|a_(m)5_|…|a_(m)12_] (mod 7) (partial components of ‘A_j_’s) are also found to meet all rules of group, ring and field (such as the existence of unique inverses for Å_j_ and an identity element) in similar manner to the original ‘A_j_’. This might mean that these smaller-sized partial illness states can be treated independently. In contrast, we can compound or identify other disease states (e.g., bipolar disorder, anxiety disorder, etc.) for the same patient if an appropriate collection of disease symptoms is accessible.

The limitations of the present study should be noted. The first is, there is no assurance that the grading scheme using the BPRS-I have equal weighting and the arbitrary scores could be calculated independently of each other. Moreover, it is unclear whether the scores we treat as components would reflect accurately states of the illness. To our knowledge, there is no evidence supporting this assumption. Grading objectively the severity of symptoms is a crucially important issue that should be determined on the bases of the data obtained and prospecting through the data employing a rigorous methodology.

Second, we developed procedures based on only ‘modulo 7 addition’ and ‘modulo 7 arithmetic’. The operations obey the rules of not only ‘group’ but also ‘ring’ and ‘field’; however, the meaning of each operation remains unclear. There is room where further development is possible. In a future study, extensions to include rings and fields (requiring multiplication and division) hopefully will have usefulness illustrated by specific applications.

Third, concerning the rules of group and others (ring, field), we assumed that ‘associativity’ is true in clinical treatment settings and/or natural developments over the course of time. Apparently, ‘commutativity’ cannot be met between medical states (e.g., the ‘A_j_’s) because changes in order of clinical treatments do cause differences in the results of disease states. Medical states and medical treatments (containing natural changes) might arise naturally though for ‘associativity’ because division into a series of treatments does not change clinical results when these would have been performed in that order; the implication being that all pertinent treatment has been provided to the patient in a specified order. For this reason, the strict algebraic structure might be the semi-group where only associativity ‘(a*b)*c = a*(b*c)’ is satisfied and inverses are not defined. Therefore, the rules of ‘ring’ and ‘field’ might not strictly hold in a clinical setting as ‘additive commutativity’, that is needed to establish a ‘ring’ and ‘field’ structure, is not present in our model. This fact might restrict such applications to some degree.

Fourth, because the ideally healthy state was in our model expressed as an identity element E, the ideally desirable treatment (with natural changes also being taken into accounted) for the state A_j_ is the operation A_j_^-1^. However, although this concept might be useful in understanding the meaning of ‘ideal treatment’, this might not be always effective as the most desirable treatment and/or clinical course. Interpreting the relationship between A_j_ and A_j_^-1^ is still unclear and does not warrant excessive examination at this stage.

Fifth and finally, our model in its present inception might be too abstract to apply to clinical scenarios. Strange to say, but we did not always put an important meaning on the total score of the BPRS-I. Conventionally, the BPRS has been assumed to be an evaluation scale for which the purpose was to sum up the individual score of each symptom. However, in this article, we exploited the psychiatric evaluation scale not only as a tool for indicating the total severity of the illness but also that as a ‘vector-based model’ (Cartesian products of modulo groups) to display the respective scores of each symptom from patient assessments. This latter interpretation has been the focus up to this point. Leastwise, the usefulness in introducing group theory and other algebraic structures in classifying severity-based expressions (grading) of disease states depends upon further improvements. Of course, the focus of the present model should be on how we can interpret the group theory structure in light of medical assessment, progression, and treatment, although that may be tenuous as this stage. We hope future studies demonstrate the utility of our method.

Without doubt, the model we have presented is far from being of immediate practical application in clinical settings. Nonetheless, we would emphasize that the versatility of the use of group theory is currently beyond imagination for reasons that behaviors of certain models can be treated generally and unexceptionally if genuine mathematical methods are performed on these models. Therefore, we heartily desire that future studies of such issues can be explored in more effective forms, not only in the field of psychiatry, but also in other medical fields, where more generalized and sophisticated operations could make a significant contribution.

## Conclusions

Within the large limitations of our methodology, it was considered that, not only in the field of psychiatry but also other fields of medicine, there might be room for a more generalized notation from the theory of groups (if not rings and fields) realized via some graduated expression. Such notation might possess potential versatility in formulating or systematizing a more rationalized descriptive model of clinical medicine that would follow similar developments in other branches of natural science.

## Appendix A

For any ‘a’ (∈Z_7_) other than ‘0’, the relation ‘a × Z_7_ (mod 7) = {a × 0, a × 1, a × 2, a × 3,…, a × 6} = Z_7_ (mod 7)’ is true. Thus, because ‘7’ (the group order of Z_7_) is a prime number, there is a unique ‘a^-1^’ (∈Z_7_) such that ‘a (mod 7) × a^-1^ (mod 7) = 1 (mod 7)’. Therefore, we can define modulo 7 division as ‘b/a (mod 7) (a,b∈Z_7_) = b × a^-1^ (mod 7)’ by using the previously defined ‘a^-1^.’ For instance, with b = 4 and a = 5 (mod 7), only one ‘a^-1^’ exists that satisfies ‘a × a^-1^ (mod 7) = 1 (mod 7)’. As for ‘5 × a^-1^ (mod 7) = 1 (mod 7)’, then ‘a^-1^ = 3’ meets the purpose. Hence, ‘b/a (mod 7)’ = 4 × 3 (mod 7) = 5 (mod 7).

## Appendix B

For example, if a certain patient is assessed under the BPRS-I, the combination of individual score ‘A_1_’ would be expressed as follows:

(17) $${\text{A}}_{1}=[1\left|4\right|0|\ldots \left|3\right|\ldots \left|6\right|1]\left(\text{mod }7\right)\text{.}$$

Hence, if the state of the same patient is reassessed some time later, the combination of the respective scores ‘A_2_’ would be described in the same way:

(18) $${\text{A}}_{2}=[2\left|6\right|3|\ldots \left|1\right|\ldots |0|5]\left(\text{mod }7\right)\text{.}$$

Subsequent patient assessments would be written in the form:

(19) $${\text{A}}_{\text{m}}{\text{ = [a}}_{\left(\text{m}\right)1}{\text{|a}}_{\left(\text{m}\right)2}{\text{|a}}_{\left(\text{m}\right)3}{\text{|…|a}}_{\left(\text{m}\right)\text{i}}{\text{|…|a}}_{\left(\text{m}\right)\text{n}-1}{\text{|a}}_{\left(\text{m}\right)\text{n}}]\left(\text{mod }7\right)$$

## Appendix C

In analogy, ‘positional vectors’ have a double meaning: one is the position itself in the vector space, and the other is as the operation of parallel translation from the origin. From this perspective, we see at once that ‘a_(m)i_’, signifying the i-th symptom of the m-th state as rated under the BPRS-I (the former meaning) can also be regarded as an operator that changes the state of the respective symptom ‘i’ from the healthy state ‘0’ to ‘a_(m)i_’ (the latter meaning). Using modulo 7 addition

(20) $${\text{a}}_{\left(\text{m}\right)\text{i}}=0\left(\text{mod 7}\right)+{\text{a}}_{\left(\text{m}\right)\text{i}}\left(\text{mod 7}\right).$$

This ensures that ‘A_m_’ can be interpreted as the operation of ‘A_m_’ on ‘E’. More generally

(21) $${\text{a}}_{\left(\text{j}\right)\text{i}}\left(\text{mod 7}\right)+{\text{a}}_{\left(\text{m}\right)\text{i}}\left(\text{mod 7}\right)={\text{a}}_{\left(\text{k}\right)\text{i}}\left(\text{mod 7}\right).$$

so that the two components a_(j)i_ and a_(k)i_ can be regarded as being linked by the operation a_(m)i_ (mod 7) = a_(k)i_ (mod 7) - a_(j)i_ (mod 7).

## Appendix D

Іb) Associativity: for any A_j_ (∈ M), the relation

(22) $$({\text{A}}_{\text{j}}*{\text{A}}_{\text{k}})*{\text{A}}_{\text{l}}={\text{A}}_{\text{j}}*({\text{A}}_{\text{k}}*{\text{A}}_{\text{l}})(\text{j},\text{ k},\text{ l}=1,2,3,\ldots ;\text{ positive integers})\text{,}$$

that is, {(A_j_ + A_k_) + A_l_} (mod 7) = {A_j_ + (A_k_ + A_l_)} (mod 7) holds.

IIb) Identity: for any A_j_ (∈ M), there exists an identity element E(=[e|e|e|…|e|…|e|e] = [0|0|0|…|0|…|0|0] (∈ M)), such that

(23) $${\text{A}}_{\text{j}}*\text{E}=\text{E}*{\text{A}}_{\text{j}}={\text{A}}_{\text{j}\text{,}}$$

that is {A_j_ + E} (mod 7) = {E + A_j_} (mod 7) = A_j_ (mod 7) holds.

IIIb) Inverse: For any A_j_ (∈ M) there exists unique element (A_j_) (∈ M), such that A_j_*(A_j_) = (A_j_)*A_j_ = E,

that is, {A_j_ + (A_j_)} (mod 7) = {(A_j_) + A_j_} (mod 7) = E (mod 7)

for (A_j_) (denote ‘A_j_^-1^’), by choosing (A_j_) as meets following relationship;

(24) $$\begin{array}{l} ({\text{A}}_{\text{j}})=[7\left|7\right|7|\ldots \left|7\right|\ldots \left|7\right|7]-{\text{ A}}_{\text{j}}\left(\text{mod }7\right)\\ =[7\left|7\right|7|\ldots \left|7\right|\ldots \left|7\right|7]-[{\text{a}}_{\left(\text{j}\right)1}|{\text{a}}_{\left(\text{j}\right)2}|{\text{a}}_{\left(\text{j}\right)3}|\ldots |{\text{a}}_{\left(\text{j}\right)\text{i}}|\ldots |{\text{a}}_{\left(\text{j}\right)\text{n}-1}|{\text{a}}_{\left(\text{j}\right)\text{n}}]\left(\text{mod }7\right) \end{array}$$

this relationship would be satisfied.

## Appendix E

All changes between the two states are included in the operator A_(j→k)_ (mod 7). From this standpoint, the change ‘A_j_ → A_k_ (mod 7)’ is encoded in the ‘operator A_(j→k)_ (mod 7)’ and would contain the patient’s response to medical treatment as graded under the BPRS (BPRS-I); natural changes accompanied in the course of the disease would automatically be included. By definition, ‘A_(j→k)_ (mod 7)’ also belongs to group ‘M’ (i.e., A_(j→k)_ ∈ M) and thus operations between any two are closed within ‘M’. Therefore, all operations among A_j_, A_k_ and A_(j→k)_, satisfy the group postulates of ‘M.’

## Appendix F

Consider state A_1_ (A.1) changing into state A_2_ (A.2), ‘A_1_ (mod 7) → A_2_ (mod 7)’; the difference operator A_(1→2)_ (mod 7) is calculated retrospectively given states A_1_ and A_2_ as follows:

(25) $$\begin{array}{l} {\text{A}}_{\left(1\to 2\right)}\left(\text{mod 7}\right)={\text{A}}_{2}\left(\text{mod 7}\right)-{\text{ A}}_{1}\left(\text{mod 7}\right)=\left\{{\text{A}}_{2}-{\text{ A}}_{1}\right\}\;\left(\text{mod 7}\right)\\ =\{[2\left|6\right|3|\ldots \left|1\right|\ldots \left|0\right|5]-[1\left|4\right|0|\ldots \left|3\right|\ldots \left|6\right|1]\}\left(\text{mod }7\right)\\ =[2-1\left|6-4\right|3-0|\ldots \left|1-3\right|\ldots \left|0-6\right|5-1]\left(\text{mod }7\right)\\ =[1\left|2\right|3|\ldots \left|-2\right|\ldots \left|-6\right|4]\left(\text{mod 7}\right)\\ =[1\left|2\right|3|\ldots \left|7-2\right|\ldots \left|7-6\right|4]\left(\text{mod }7\right)\\ =[1\left|2\right|3|\ldots \left|5\right|\ldots \left|1\right|4]\left(\text{mod }7\right)\text{.} \end{array}$$

The component ‘-2’ is equivalent to ‘5’, and ‘-6’ is also to ‘1’ under the condition of modulo 7 operation. Operationally, the use of the minus ‘-’ is convenient in understanding decreases in the severity of symptoms, occurring when the value of the component of the latter state is smaller than that of the former state; if ‘5’ → ‘3’, then ‘3-5’ = ‘-2’ = ‘5’ under modulo 7 addition (subtraction).

We verify relation (4) for A_1_ and A_(1→2)_ in the following way:

(26) $$\begin{array}{l} {\text{A}}_{1}*{\text{A}}_{\left(1\to 2\right)}={\text{A}}_{1}\left(\text{mod 7}\right)+{\text{A}}_{\left(1\to 2\right)}\left(\text{mod 7}\right)=\left\{{\text{A}}_{1}+{\text{A}}_{\left(1\to 2\right)}\right\}\;\left(\text{mod }7\right)\\ =\{[1\left|4\right|0|\ldots \left|3\right|\ldots \left|6\right|1]+[1\left|2\right|3|\ldots \left|5\right|\ldots \left|1\right|4]\}\left(\text{mod }7\right)\\ =[1+1\left|4+2\right|0+3|\ldots \left|3+5\right|\ldots \left|6+1\right|1+4]\left(\text{mod }7\right)\\ =[2\left|6\right|3|\ldots \left|1\right|\ldots \left|0\right|5]\left(\text{mod }7\right)\\ ={\text{ A}}_{2}\left(\text{mod }7\right) \end{array}$$

## Appendix G

For multiplication of two elements

(27) $$\begin{array}{l} {\text{A}}_{\text{j}}\left(\text{mod 7}\right)\times {\text{A}}_{\text{k}}\left(\text{mod 7}\right)=({\text{A}}_{\text{j}}\times {\text{A}}_{\text{k}})\left(\text{mod 7}\right)\\ =[{\text{a}}_{\left(\text{j}\right)1}\times {\text{a}}_{\left(\text{k}\right)1}|{\text{a}}_{\left(\text{j}\right)2}\times {\text{a}}_{\left(\text{k}\right)2}|{\text{a}}_{\left(\text{j}\right)3}\times {\text{a}}_{\left(\text{k}\right)3}|\ldots |{\text{a}}_{\left(\text{j}\right)\text{i}}\times {\text{a}}_{\left(\text{k}\right)\text{i}}|\ldots |{\text{a}}_{\left(\text{j}\right)\text{n}-1}\times {\text{a}}_{\left(\text{k}\right)\text{n}-1}|{\text{a}}_{\left(\text{j}\right)\text{n}}\times {\text{a}}_{\left(\text{k}\right)\text{n}}] \end{array}$$

(for all components; mod 7).

Therefore, (A_j_ × A_k_) (mod 7) ∈ M, and the group order of M is 7^18^ (= W).

Let ‘A_k_’ be an element of M other than ‘E’. Then ‘A_k_ × M (mod 7) = {A_k_ × A_1_, A_k_ × A_2_, A_k_ × A_3_,…, A_k_ × A_W_} = M (mod 7)’ is true.

Hence, (A_j_ × A_k_) (mod 7) ∈ M, as modulo 7 multiplication is closed as an operation.

Because ‘A_k_^-1^’ is composed of the n-tuple product of components from ‘a_(k)1_^-1^’ to ‘a_(k)n_^-1^’ (n = 18), the same argument holds for each component, and for any ‘A_k_’ with the exception of ‘E’ (=[0|0|0|…|0|…|0|0]). Each ‘A_k_^-1^’ (∈ M) is uniquely defined by relation ‘A_k_^-1^ = [a_(k)1_^-1^|a_(k)2_^-1^|a_(k)3_^-1^|…|a_(k)i_^-1^|…|a_(k)n_^-1^]’ with ‘a_(k)i_ (mod 7) × a_(k)i_^-1^ (mod 7) = 1 (mod 7)’;

(28) $$\begin{array}{l} {\text{A}}_{\text{k}}\left(\text{mod }7\right)\times {\text{A}}_{\text{k}}{}^{-1}\left(\text{mod }7\right)\\ =[{\text{a}}_{\left(\text{k}\right)1}\times {\text{a}}_{\left(\text{k}\right)1}{}^{-1}|{\text{a}}_{\left(\text{k}\right)2}\times {\text{a}}_{\left(\text{k}\right)2}{}^{-1}|{\text{a}}_{\left(\text{k}\right)3}\times {\text{a}}_{\left(\text{k}\right)3}{}^{-1}|\ldots |{\text{a}}_{\left(\text{k}\right)\text{i}}\times {\text{a}}_{\left(\text{k}\right)\text{i}}{}^{-1}|\ldots |{\text{a}}_{\left(\text{k}\right)\text{n}-1}\times {\text{a}}_{\left(\text{k}\right)\text{n}-1}{}^{-1}|{\text{a}}_{\left(\text{k}\right)\text{n}}\times {\text{a}}_{\left(\text{k}\right)\text{n}}{}^{-1}]\left(\text{mod 7}\right)\\ =[1\left(\text{mod }7\right)\left|1\left(\text{mod }7\right)\right|1\left(\text{mod }7\right)|\ldots \left|1\left(\text{mod }7\right)\right|\ldots |1\left(\text{mod }7\right)|1\left(\text{mod }7\right)]\\ =[1\left|1\right|1|\ldots \left|1\right|\ldots |1]\left(\text{mod }7\right) \end{array}$$

Hence, we can define ‘A_j_ (mod 7)/A_k_ (mod 7)’ in the following way:

(29) $$\begin{array}{l} {\text{A}}_{\text{j}}\left(\text{mod }7\right)/{\text{A}}_{\text{k}}\left(\text{mod }7\right)=({\text{A}}_{\text{j}}/{\text{A}}_{\text{k}})\left(\text{mod }7\right)=({\text{A}}_{\text{j}}\times {\text{A}}_{\text{k}}{}^{-1})\left(\text{mod }7\right)\\ =[{\text{a}}_{\left(\text{j}\right)1}\times {\text{a}}_{\left(\text{k}\right)1}{}^{-1}|{\text{a}}_{\left(\text{j}\right)2}\times {\text{a}}_{\left(\text{k}\right)2}{}^{-1}|{\text{a}}_{\left(\text{j}\right)3}\times {\text{a}}_{\left(\text{k}\right)3}{}^{-1}|\ldots |{\text{a}}_{\left(\text{j}\right)\text{i}}\times {\text{a}}_{\left(\text{k}\right)\text{i}}{}^{-1}|\ldots |{\text{a}}_{\left(\text{j}\right)\text{n}-1}\times {\text{a}}_{\left(\text{k}\right)\text{n}-1}{}^{-1}|{\text{a}}_{\left(\text{j}\right)\text{n}}\times {\text{a}}_{\left(\text{k}\right)\text{n}}{}^{-1}] \end{array}$$

where a_(k)i_ (mod 7) × a_(k)i_^-1^ (mod 7) = 1 (mod 7) holds.

Thus, (A_j_/A_k_) (mod 7) belongs to M, and modulo 7 division over M is closed as an operation. To sum up, division modulo 7 can be defined for G and M.

## Appendix H

The group postulates for elements of G and M are demonstrated below.

Commutative law for addition:

(30) $$\begin{array}{l} {\text{a}}_{\left(\text{j}\right)\text{i}}\left(\text{mod }7\right){\text{ + a}}_{\left(\text{k}\right)\text{i}}\left(\text{mod }7\right){\text{ = \{a}}_{\left(\text{j}\right)\text{i}}{\text{ + a}}_{\left(\text{k}\right)\text{i}}\text{\} }\left(\text{mod }7\right)\\ {\text{= \{a}}_{\left(\text{k}\right)\text{i}}{\text{ + a}}_{\left(\text{j}\right)\text{i}}\text{\} }\left(\text{mod }7\right){\text{ = a}}_{\left(\text{k}\right)\text{i}}\left(\text{mod }7\right){\text{ + a}}_{\left(\text{j}\right)\text{i}}\left(\text{mod }7\right)\\ {\text{A}}_{\text{j}}\left(\text{mod }7\right){\text{ + A}}_{\text{k}}\left(\text{mod }7\right){\text{ = \{A}}_{\text{j}}{\text{ + A}}_{\text{k}}\text{\} }\left(\text{mod }7\right)\\ {\text{= \{A}}_{\text{k}}{\text{ + A}}_{\text{j}}\text{\} }\left(\text{mod }7\right){\text{ = A}}_{\text{k}}\left(\text{mod }7\right){\text{ + A}}_{\text{j}}\left(\text{mod }7\right) \end{array}$$

Associative law for multiplication:

(31) $$\begin{array}{l} \{{\text{a}}_{\left(\text{j}\right)\text{i}}\times {\text{a}}_{\left(\text{k}\right)\text{i}}\}\times {\text{a}}_{\left(\text{l}\right)\text{i}}\left(\text{mod }7\right)={\text{a}}_{\left(\text{j}\right)\text{i}}\times \{{\text{a}}_{\left(\text{k}\right)\text{i}}\times {\text{a}}_{\left(\text{l}\right)\text{i}}\}\left(\text{mod }7\right)\\ \{{\text{A}}_{\text{j}}\times {\text{A}}_{\text{k}}\}\times {\text{A}}_{\text{l}}\left(\text{mod }7\right)={\text{A}}_{\text{j}}\times \{{\text{A}}_{\text{k}}\times {\text{A}}_{\text{l}}\}\left(\text{mod }7\right) \end{array}$$

Distributive law for addition and multiplication:

(32) $$\begin{array}{l} {\text{a}}_{\left(\text{j}\right)\text{i}}\times \{{\text{a}}_{\left(\text{k}\right)\text{i}}+{\text{a}}_{\left(\text{l}\right)\text{i}}\}\left(\text{mod }7\right)={\text{a}}_{\left(\text{j}\right)\text{i}}\times {\text{a}}_{\left(\text{k}\right)\text{i}}+{\text{a}}_{\left(\text{j}\right)\text{i}}\times {\text{a}}_{\left(\text{l}\right)\text{i}}\left(\text{mod }7\right)\\ \{{\text{a}}_{\left(\text{j}\right)\text{i}}+{\text{a}}_{\left(\text{k}\right)\text{i}}\}\times {\text{a}}_{\left(\text{l}\right)\text{i}}\left(\text{mod }7\right)={\text{a}}_{\left(\text{j}\right)\text{i}}\times {\text{a}}_{\left(\text{l}\right)\text{i}}+{\text{a}}_{\left(\text{k}\right)\text{i}}\times {\text{a}}_{\left(\text{l}\right)\text{i}}\left(\text{mod }7\right)\\ {\text{A}}_{\text{j}}\times \{{\text{A}}_{\text{k}}+{\text{A}}_{\text{l}}\}\left(\text{mod }7\right)={\text{A}}_{\text{j}}\times {\text{A}}_{\text{k}}+{\text{A}}_{\text{j}}\times {\text{A}}_{\text{l}}\left(\text{mod }7\right)\\ \{{\text{A}}_{\text{j}}+{\text{A}}_{\text{k}}\}\times {\text{A}}_{\text{l}}\left(\text{mod }7\right)={\text{A}}_{\text{j}}\times {\text{A}}_{\text{l}}+{\text{A}}_{\text{k}}\times {\text{A}}_{\text{l}}\left(\text{mod }7\right) \end{array}$$

## Competing interests

The authors declare that they have no competing interests.

## Authors’ contributions

JS conceived of the main idea of this article and wrote the manuscript. SM revised the manuscript. JI gave advice on the potential applicability of the model to clinical research and treatment. All authors read and approved the final manuscript.
